# A Novel and Quantitative Detection Assay (*effluxR*) for Identifying Efflux-Associated Resistance Genes Using Multiplex Digital PCR in Clinical Isolates of *Pseudomonas aeruginosa*

**DOI:** 10.3390/mps6050096

**Published:** 2023-10-08

**Authors:** Nontaporn Rattanachak, Sattaporn Weawsiangsang, Robert A. Baldock, Theerasak Jaifoo, Touchkanin Jongjitvimol, Jirapas Jongjitwimol

**Affiliations:** 1 Biomedical Sciences Program, Faculty of Allied Health Sciences, Naresuan University, Phitsanulok 65000, Thailand; nontapornr63@nu.ac.th (N.R.); sattapornw63@nu.ac.th (S.W.); 2 School of Pharmacy and Biomedical Sciences, Faculty of Science and Health, University of Portsmouth, Portsmouth PO1 2DT, UK; robert.baldock@port.ac.uk; 3 Master of Science Program in Medical Technology, Faculty of Allied Health Sciences, Naresuan University, Phitsanulok 65000, Thailand; teerasak.lab@gmail.com; 4 Biology Program, Faculty of Science and Technology, Pibulsongkram Rajabhat University, Phitsanulok 65000, Thailand; 5 Department of Medical Technology, Faculty of Allied Health Sciences, Naresuan University, Phitsanulok 65000, Thailand; 6 Centre of Excellence in Biomaterials, Faculty of Science, Naresuan University, Phitsanulok 65000, Thailand

**Keywords:** *Pseudomonas aeruginosa*, antibiotic resistant genes, RND-type efflux pumps, multiplex digital polymerase chain reaction (mdPCR), multiplex quantitative polymerase chain reaction (mqPCR)

## Abstract

The rise of multidrug resistance of *Pseudomonas aeruginosa* highlights an increased need for selective and precise antimicrobial treatment. Drug efflux pumps are one of the major mechanisms of antimicrobial resistance found in many bacteria, including *P*. *aeruginosa*. Detection of efflux genes using a polymerase chain reaction (PCR)-based system would enable resistance detection and aid clinical decision making. Therefore, we aimed to develop and optimize a novel method herein referred to as “*effluxR* detection assay” using multiplex digital PCR (mdPCR) for detection of *mex* efflux pump genes in *P. aeruginosa* strains. The annealing/extension temperatures and gDNA concentrations were optimized to amplify *mexB*, *mexD*, and *mexY* using the multiplex quantitative PCR (mqPCR) system. We established the optimal mqPCR conditions for the assay (Ta of 59 °C with gDNA concentrations at or above 0.5 ng/µL). Using these conditions, we were able to successfully detect the presence of these genes in a quantity-dependent manner. The limit of detection for *mex* genes using the *effluxR* detection assay with mdPCR was 0.001 ng/µL (7.04–34.81 copies/µL). Moreover, using blind sample testing, we show that *effluxR* detection assay had 100% sensitivity and specificity for detecting *mex* genes in *P*. *aeruginosa*. In conclusion, the *effluxR* detection assay, using mdPCR, is able to identify the presence of multiple *mex* genes in *P*. *aeruginosa* that may aid clinical laboratory decisions and further epidemiological studies.

## 1. Introduction

*Pseudomonas aeruginosa* is a Gram-negative bacteria which is recognized as an opportunistic pathogen, causing community and nosocomial infections with a high mortality rate [1,2,3]. The mortality rate from nosocomial pneumonia caused by *P. aeruginosa* is estimated to be around 70% [4]. Mortality arising from septicemia with *P. aeruginosa* has been estimated at between 30–50% [5]. Moreover, *P. aeruginosa* is highly adaptable to environmental changes, due, in part, to its large genome size (5.5–7.0 million base pairs), which encodes a significant number of enzymes regulating metabolism, transportation, and organic compound efflux [6,7]. These mechanisms enable P. aeruginosa to survive in a wide range of environments and spread worldwide [6,7]. Furthermore, these mechanisms permit the resistance of *P. aeruginosa* to various antimicrobial agents, thereby contributing to the development of multidrug resistance (MDR) [8,9,10,11]. MDR in *P. aeruginosa* is challenging to treat and results in patient mortality rates of about 15–30% in some regions [12,13,14]. *P. aeruginosa* is one of three bacterial species which presents a significant health concern worldwide [15,16]. A rapid diagnostic method for identifying drug-resistant *P. aeruginosa* infections is essential to reduce inappropriate antimicrobial use and, by enabling the selection of the most appropriate treatment initially, reducing mortality rates [17].

A mechanism used by *P*. *aeruginosa* to confer antibiotic resistance is efflux pumps, which transport antibiotics or compounds through the outer membrane and out of the cell [18]. The resistance-nodulation-division (RND) efflux pump mechanism of *P. aeruginosa* is known to enable multidrug resistance [19]. At least three crucial RND pumps, namely the MexAB-OprM, MexCD-OprJ, and MexXY-OprA systems, contribute significantly to antibiotic resistance [18,20,21]. Commonly, the RND pump forms a tripartite complex, involving the outer membrane (via OprM, OprJ, or OprA), the inner membrane (via MexB, MexD, MexF, or MexY), and the periplasmic space components (MexA, MexC, and MexX). Antibiotics or compounds are pumped out of the cell via the coordinated action of the three protein components [22]. An outer membrane protein facilitates the transfer of the substrate through the outer membrane. The inner membrane portion is responsible for substrate specificity and also facilitates pH-related drug transport. The periplasmic space fusion protein bridges the inner and outer membrane proteins [23]. The actions of many antibiotic classes, such as β-lactams, aminoglycosides, fluoroquinolones, tigecycline, cephalosporins, chloramphenicol, and tetracyclines, are negated through the activity of RND pumps [22,24,25].

A polymerase chain reaction (PCR)-based system can be used to detect genes associated with antibiotic resistance [26,27,28]. Successive generations of the PCR systems have enabled higher resolution and more rapid detection of genes and even specific polymorphisms. Conventional PCR enables qualitative gene detection using gel electrophoresis for end-point analysis. Second-generation systems (i.e., real-time or quantitative PCR (qPCR)), generate results using a standard curve by monitoring real-time amplification with fluorescence dyes. Quantitative PCR has been effectively used for the detection of various pathogenic microorganisms, including viruses, bacteria, and fungi [1,2]. Furthermore, this technique has proven useful in the quantitative detection of pathogenic organisms in clinical settings [3]. In qPCR, results are obtained from the cycle threshold (CT), the number of cycles at which the fluorescent signal rises above the threshold set by controls. The latest generation of systems, digital PCR (dPCR), obtains the end-point result as the absolute quantification of nucleic acid concentration using Poisson statistical analyses [29]. Moreover, dPCR is a robust, highly sensitive technique with outstanding repeatability and no requirement for a standard curve [29].

Detecting the presence of efflux-associated resistance genes is paramount for informing the choice and dosage of antibiotics to treat patients clinically as well as providing potentially valuable epidemiological information [30]. While dPCR has been widely used in various applications, its specific application for detecting efflux pump genes in bacteria is limited. We hypothesized that if a novel and effective dPCR method was available for detecting efflux pump genes, it would aid the detection of drug resistance in clinical *P. aeruginosa* isolates. Therefore, this study aimed to develop and optimize a novel methodology herein named the “*effluxR* detection assay” using a multiplex digital PCR (mdPCR) system to detect the three main efflux pump genes (*mexB*, *mexD*, and *mexY*) in *P*. *aeruginosa*. Here, we have optimized the PCR conditions using a probe-based multiplex quantitative PCR system (mqPCR) for detection of resistance genes in *P*. *aeruginosa*. We found that the *effluxR* detection assay is highly sensitive and specific for identifying the presence of *mexB*, *mexD*, and *mexY* in *P*. *aeruginosa* from clinical isolates.

## 2. Materials and Methods

### 2.1. Bacterial Strains and Growth Conditions

The bacterial reference strains, namely *Pseudomonas aeruginosa* ATCC27853, *P. aeruginosa* ATCC BAA-2108, *Staphylococcus aureus* ATCC29213, *S. aureus* ATCC25923, *Escherichia coli* ATCC25922, *E. coli* ATCC2452, *Klebsiella pneumoniae* ATCC1705, and *Enterobacter cloacae* ATCC2341 were purchased from American Type Culture Collection (ATCC). Clinical isolates (PA.CI) of *P. aeruginosa* strains were received from hospitalized patients at Kamphaeng Phet Hospital, Kamphaeng Phet, Thailand in 2022. Depending on the particular experiments, tryptone soya agar (TSA; Oxoid, UK) and/or tryptone soya broth (TSB; Oxoid, UK) were used to grow each bacterium tested in this study. All the bacterial cultures were incubated at 35 ± 2 °C for 18–24 h. The turbidity of the inoculum in each experiment was equivalent to a 0.5 McFarland standard (1–2 × 10^8^ CFU/mL) by adjustment with a densitometer (Biosan, Riga, Latvia).

### 2.2. Genomic DNA Extraction

All bacterial cells grown in TSB were harvested at 24 h by centrifugation. A Genomic DNA Isolation Kit (Bio-Helix, Taipei, Taiwan) was used to purify bacterial genomic DNA (gDNA) according to the manufacturer’s protocol. Briefly, the pelleted cells were lysed using the lysis buffer. The samples were then vortexed and incubated at 60 °C for 10 min. RNA was removed by adding 10 mg/mL RNase A at room temperature for 5 min. To remove protein, the protein buffer was added to the sample tubes. The samples were then centrifuged at 12,000 rpm for 1 min. Each supernatant was then collected and transferred to a 2 mL collection tube. After centrifugation, the washing buffer was added in the tube to wash the sample twice. The gDNA sample was eluted from the column with 50 µL DNase-free water and the tube was then centrifuged at 14,000 rpm for 2 min. The gDNA concentration and purity were verified using a Calibri Microvolume Spectrophotometer (Titertek-Berthold, Pforzheim, Germany).

### 2.3. Detection of the Mex Efflux Pump Genes Using Multiplex Quantitative PCR (mqPCR)

The isolated gDNA was used as a template to detect the *mex* genes using probe-based mqPCR. The *16S rRNA* gene was used as a reference. The *mex* genes were then amplified using specific primers and probes (Table 1). The cycle threshold (CT) value was reported by measuring the change in fluorescence with each cycle. In each run, all tests were performed in triplicate with a non-template control (NTC). This approach was used to determine the optimal conditions for the *effluxR* detection assay.

#### 2.3.1. Investigation of Optimal mqPCR Annealing/Extension Temperature

Five temperatures ranging from 58 to 62 °C were tested. The mqPCR reactions were prepared following the manufacturer’s guidance for the QIAcuity Probe PCR Kit (QIAGEN, Hilden, Germany). Each reaction contained 5 ng of the gDNA template, 0.8 µM of each forward and reverse primer, 0.4 µM of each probe (Table 1), 5 µL of PCR Master Mix and RNase-free water up to a total volume of 20 µL. After mixture preparation, the LineGene 9600 Plus Real-Time PCR Detection System (Bioer Technology, Hangzhou, China) was used to analyze the *mex* efflux pump genes in *P. aeruginosa* strains. The following thermal cycler conditions were used: initial denaturation at 95 °C for 2 min; 40 cycles of denaturation at 95 °C for 20 s; and combined annealing/extension at tested temperatures for 30 s.

#### 2.3.2. Investigation of Optimal gDNA Concentration of *P. aeruginosa* Strains

Different gDNA concentrations were investigated for amplification of representative *mex* efflux pump genes in *P. aeruginosa* strains. To prepare the reaction, gDNA concentrations of 5.0, 2.5, 1.0, and 0.5 ng/µL were added into a PCR tube containing the same PCR reagents as above. The thermal cycler conditions were as follows: first denaturation at 95 °C for 2 min; 40 cycles of denaturation at 95 °C for 20 s; and combined annealing/extension at the optimal temperature for 30 s.

### 2.4. DNA Agarose Gel Electrophoresis

The expected sizes of amplicons (Table 1) were checked on 2% agarose electrophoresis gels. Briefly, 2% agarose (GeneDireX, Inc., Taoyuan, Taiwan) was prepared in Tris-borate-EDTA (TBE) buffer containing 0.5 mg/mL of UltraPure™ Ethidium Bromide (EtBr) (Cat. No. 15585011, Thermo Fisher Scientific, Waltham, MA, USA). A mixture of 10 µL PCR samples with 2 µL of a 6× loading buffer (Cat. No. LD001–1000, GeneDireX, Inc., Taoyuan, Taiwan) was loaded into the well. A DNA marker was also loaded as reference sizes (Cat. No. DM011-R500, Kplus DNA Ladder RTU, GeneDireX, Inc., Taoyuan, Taiwan). The gels were run on a Bio-Rad electrophoresis system (Bio-Rad Laboratories, Inc., Hercules, CA, USA) at constant 120 V for 30–40 min. The bands on the gels were visualized under a UV light mode of a gel documentation system (Aplegen, Ramsey, NJ, USA).

### 2.5. Development of the EffluxR Detection Assay to Detect the mex Efflux Pump Genes Using Multiplex Digital PCR (mdPCR)

**Figure 1:**
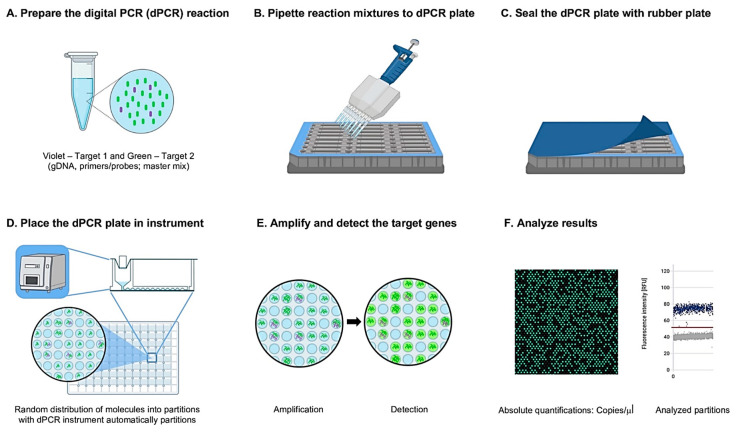
The schematic amplification of target genes using digital PCR.

In this study, we developed the *effluxR* detection assay to simultaneously detect the presence of *mex* efflux pump genes from the bacterial samples using the multiplex QIAcuity Digital PCR system (QIAGEN, Hilden, Germany). A QIAcuity Probe PCR Kit (QIAGEN, Hilden, Germany) was used for mdPCR reactions, following the manufacturer’s instructions. Briefly, all mdPCR reactions were performed at a final volume of 40 µL. Each reaction contained different quantities of gDNA (as indicated), 0.8 µM of each forward and reverse primer, 0.4 µM of each probe (Table 1), and 10 µL of PCR Master Mix and RNase-free water. The mdPCR reactions were then pipetted to a 24-well QIAcuity Nanoplate (QIAGEN, Hilden, Germany). The nanoplate was sealed with a rubber sheet and loaded in the QIAcuity Digital PCR instrument (QIAGEN, Hilden, Germany) (Figure 1). The thermal cycler conditions of the mdPCR system were as follows: 40 cycles of denaturation at 95 °C for 15 s and combined annealing/extension at 59 °C for 30 s. Fluorescence measurements were then made for each reaction. A non-template control (NTC) was used as a negative control. All analyzable partitions, including the valid, positive, and negative partitions, were used to calculate the total number of copies of the target molecule (λ) in all valid partitions (copies per partition) according to Equation (1). The absolute quantification (λ_volume_ as the unit of copies/µL) was then calculated as shown in Equation (2), where the estimated partition volume (V) is 0.91 nL for the 26k-nanoplate system.

(1) $$\lambda =-\ln\;\left(\frac{\mathrm{Number}\;\mathrm{of}\;\mathrm{valid}\;\mathrm{partitions}-\mathrm{Number}\;\mathrm{of}\;\mathrm{positive}\;\mathrm{partitions}}{\mathrm{Number}\;\mathrm{of}\;\mathrm{valid}\;\mathrm{partitions}}\right)$$

(2) $$\lambda_{\mathrm{volume}}=\frac{\lambda }{V\;[µL]}$$

#### 2.5.1. Optimization of gDNA Concentration in *P. aeruginosa*

To determine the gDNA concentration using *effluxR* detection assay with the mdPCR system, a series of known concentrations of gDNA concentrations (5.00, 2.50, 1.00, 0.50, and 0.05 ng/µL) were analyzed under the optimal conditions obtained from mqPCR. The presented results were interpreted as a positive partition percentage.

#### 2.5.2. Limit of Detection (LOD) of the *effluxR* Detection Assay Using the mdPCR System

To investigate the LOD of the *effluxR* detection assay using the mdPCR system, the gDNA concentrations of the ATCC27853 strain were diluted with RNase-free water to achieve various concentrations, including 2.50, 1.25, 0.50, 0.05, 0.005, 0.003, 0.001, and 0.000 ng/µL. The *mex* genes were detected as mentioned above and then interpreted as the absolute quantification in the unit of copies/µL. The 95% confidence interval (CI) in copies/µL is also reported following the manufacture’s calculation (QIAGEN, Hilden, Germany).

#### 2.5.3. Sensitivity and Specificity Determination of the *effluxR* Detection Assay Using the mdPCR System

To determine the sensitivity and specificity of the *effluxR* detection assay with the mdPCR system, a blind test was carried out to detect 69 positive-*mex* efflux pump gene samples and 15 negative samples. In brief, blinded gDNA samples were mixed with a PCR probe mixture following the manufacturer’s instructions. The *mexB*, *mexD*, and *mexY* genes were interpreted as the percentage of positive partitions.

### 2.6. Statistical Analysis

The mean differences of the CT values from mqPCR were analyzed using Kruskal–Wallis one-way analysis of variance (ANOVA) in GraphPad Software (GraphPad Prism version 8.2.0, San Diego, CA, USA). Both mean CT values and standard deviations are reported. For the mdPCR system, the results were reported as the absolute quantification of the target genes, based on the number of positive and negative partitions. The correlation between absolute quantification and gDNA concentrations was analyzed using a Pearson’s correlation (two-tailed) using GraphPad Software. ROC analysis using IBM SPSS software version 23 (IBM Corp., Armonk, NY, USA) and the Youden’s index (J) were used for the validation of the cut-off values.

## 3. Results

### 3.1. Optimising Annealing/Extension Temperatures for Amplifying the RND Genes Using mqPCR

To amplify representative *mex* efflux pump genes of *P. aeruginosa* strains, the optimal mqPCR annealing/extension temperature of the genes needed to be determined for *P. aeruginosa*. A range of annealing/extension temperatures were tested using mqPCR including 58 °C, 59 °C, 60 °C, 61 °C, and 62 °C. Resulting cycle thresholds (CT) of *mexB*, *mexD*, and *mexY*, as well as the reference gene (*16s rRNA*) from both strains, are shown in Table 2. CT values ranged from 13.17–23.17 cycles. The results showed that the *mexB* gene was detected in both strains at the different annealing/extension temperatures, with the CT values ranging from 16.47–23.17 cycles. For the *mexD* gene, the CT value ranged from 15.57 to 17.66 cycles at the temperatures tested. The CT values of the *mexY* and *16s rRNA* genes were detected at 15.38–17.42 and 13.17–18.92 cycles, respectively, at the temperatures tested. Overall, the result showed that all annealing/extension temperatures tested in this study were not statistically different (all *p*-values of > 0.9999), indicating that *mexB*, *mexD*, and *mexY* genes successfully amplified in *P. aeruginosa* ATCC27853 and *P. aeruginosa* ATCC BAA-2108 between 58 and 62 °C.

### 3.2. Optimal gDNA Concentration of P. aeruginosa ATCC for Amplifying the Mex Genes Using mqPCR

To optimize the gDNA concentrations for the amplification of the *mex* efflux pump genes using the mqPCR system, we used the optimal annealing/extension temperature of 59 °C. All gDNA concentrations tested (5.0, 2.5, 1.0, and 0.5 ng/µL) showed the CT values ranging from 15.49–27.31 cycles of all *mexB*, *mexD*, *mexY*, and *16s rRNA* genes (Table 3). For *mexB* of both *P. aeruginosa* strains, the CT values of gDNA concentrations tested ranged from 17.97–27.31 cycles. In both *P. aeruginosa* genome samples, the CT values of *mexD* were shown in the range from 19.80–22.93 cycles at the gDNA concentrations tested. At the same time, the *mexY* gene of both *P. aeruginosa* strains tested showed CT values ranging from 18.98–22.17 cycles. For the reference gene, *16s rRNA*, at gDNA concentrations tested, the CT value was shown in the range from 15.49–21.04 cycles (Table 3). This result indicated that the gDNA samples of *P. aeruginosa* ATCC27853 and *P. aeruginosa* ATCC BAA-2108 in all concentrations tested had strong positive reactions, indicating that they are suitable for detection of the *mex* efflux pump genes in these samples.

### 3.3. MexB, mexD, mexY, and 16s rRNA Bands were Detected in All P. aeruginosa Strains Using Agarose Gel Electrophoresis

**Figure 2:**
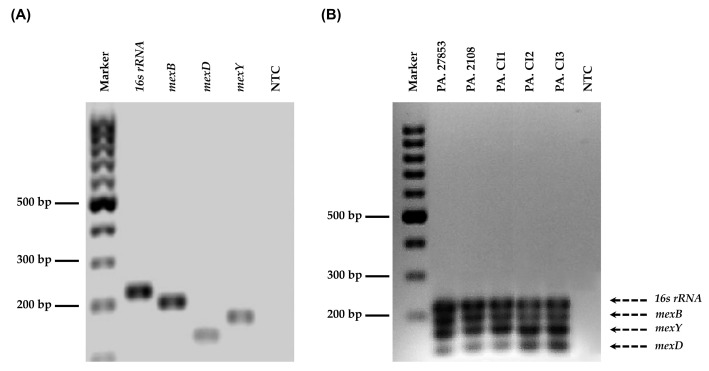
Agarose gel electrophoresis analysis of the reference (*16s rRNA*) and *mex* efflux pump genes (*mexB*, *mexD*, and *mexY*) amplified with (**A**) singleplex PCR system from representative *P*. *aeruginosa* ATCC 27853 strains and (**B**) multiplex PCR system from five representative *P. aeruginosa* strains. The PCR products amplified were run on a 2% agarose gel. The gDNA concentration was 5 ng/µL (the gDNA amount was 5 ng). The first lane contains a DNA ladder of fragments of known sizes. “PA. 27853” is the sample from *P. aeruginosa* ATCC27853. “PA. 2108” is the sample from *P. aeruginosa* ATCC BAA-2108. “PA. CI1, PA. CI2, and PA. CI3”, are the samples from *P. aeruginosa* clinical isolate no. 1, no. 2, and no. 3, respectively. NTC denotes the non-template control.

We sought to verify that the amplicons generated using the optimized mqPCR conditions were of the anticipated size by agarose gel electrophoresis (Figure 2B). Genomic DNA (5 ng) were amplified at 59 °C with the *mex* and reference primer sets. The results showed that the amplicons of the reference gene, *16s rRNA*, in all *P. aeruginosa* gDNA samples were of the expected size of 225 bp (Figure 2A,B; Table 1). The amplicons for the *mexB, mexD,* and *mexY* genes were shown near 199 bp, 131 bp, and 168 bp, respectively (Figure 2A,B), as expected (Table 1). The results confirmed that the optimal conditions for mqPCR (including the annealing/extension temperatures, concentration of the gDNA and specific primer sets) were suitable for amplifying the drug resistance genes in the *P. aeruginosa* strains tested.

### 3.4. The Mex Efflux Pump Genes Can Be Detected at a Range of gDNA Concentrations in P. aeruginosa Using the effluxR Detection Assay with the mdPCR System

**Figure 3:**
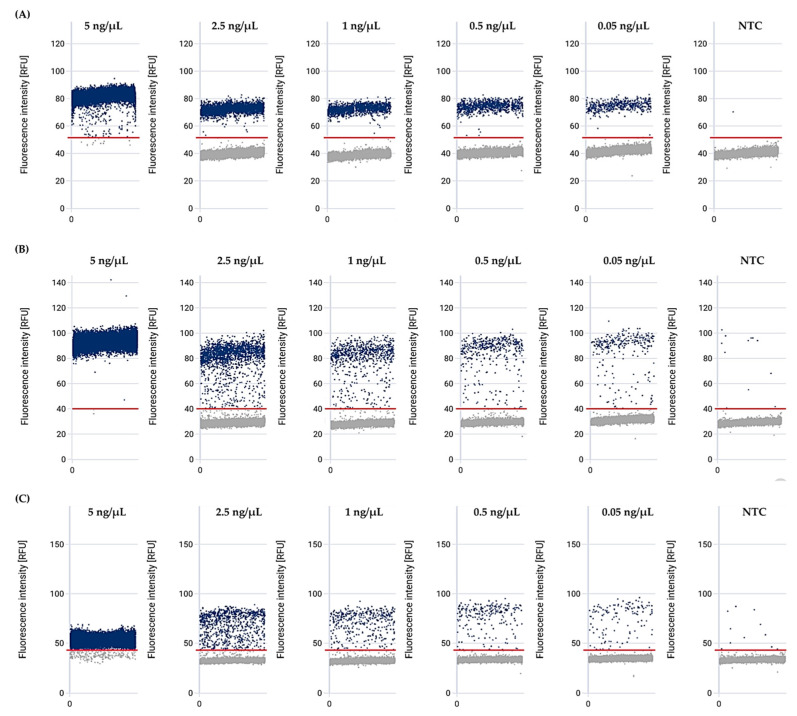
The fluorescence intensity of *mex* efflux pump genes at various gDNA concentrations of *P*. *aeruginosa* ATCC27853, detected by multiplex digital PCR, for (**A**) *mexB*, (**B**) *mexD*, and (**C**) *mexY*. Abbreviation; RFU: relative fluorescence units, NTC: Non-template control. The red lines represent the fluorescence threshold. Blue dots above the threshold line are considered as positive partitions, whereas grey dots below the threshold line are considered as negative partitions.

**Figure 4:**
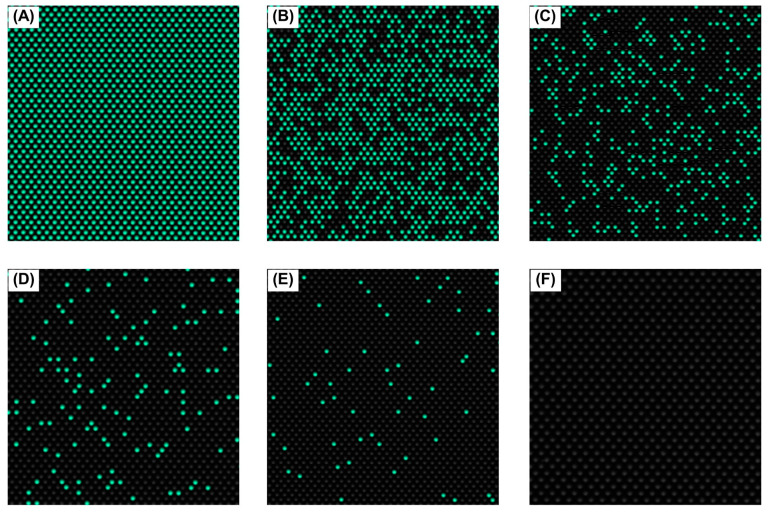
The validated microscopic images of *mexB* gene as the representative for positive reactions (**A**–**E**) and a negative reaction (**F**) in various gDNA concentrations of *P. aeruginosa* ATCC27853: (**A**) 5 ng/µL; (**B**) 2.5 ng/µL; (**C**) 1.0 ng/µL; (**D**) 0.5 ng/µL; (**E**) 0.05 ng/µL; and (**F**) non-template. The green dots represent the positive partitions containing the target genes.

We examined the presence of representative *mex* efflux pump genes, including *mexB*, *mexD*, and *mexY* of *P. aeruginosa* ATCC27853, at various gDNA concentrations using the *effluxR* detection assay with the mdPCR system. The positive and negative partitions of each *mex* gene in *P. aeruginosa* were presented as the relative fluorescent intensity units on a 1D scatter plot (Figure 3). At the same time, microscopic image validation was used to identify the specific *mex* target genes using specific fluorescent dyes in the filled partitions (Figure 4). The aggregated results of all three replicates demonstrated a concentration-dependent signal with strong fluorescence intensities of all genes tested, which would enable absolute quantification of gene copies. For *P. aeruginosa* gDNA concentrations of 5.00 ng/µL*,* the positive partitions of *mexB*, *mexD*, and *mexY* genes were 100%. At a gDNA concentration of 2.50 ng/µL, the positive partitions were 100%, 100%, and 96.33% for *mexB*, *mexD*, and *mexY*, respectively, while the positive partitions of *mexB*, *mexD*, and *mexY* with 1.00 ng/µL of gDNA were 99.33%, 97.00% and 82.00%, respectively. For *P. aeruginosa* gDNA concentrations of 0.50 ng/µL, the positive partitions were 88.67%, 84.67%, and 72.33% for *mexB*, *mexD*, and *mexY*, respectively. Finally, with 0.05 ng/µL gDNA concentration, the positive partitions were 58.00%, 36.67%, and 31.67% for *mexB*, *mexD*, and *mexY*, respectively.

### 3.5. Detection Limit of the effluxR Detection Assay with the mdPCR System Is 0.001 ng/µL Equivalent to 7.04 copies/µL of the Mex Efflux Pump Genes in P. aeruginosa ATCC27853

The limit of detection (LOD) for the *effluxR* detection assay using the mdPCR platform was determined by detecting the presence of representative *mex* efflux pump genes at a range of genomic concentrations of the *P. aeruginosa* strain, ATCC27853. Concentrations assayed include 2.50 ng/µL, 1.25 ng/µL, 0.50 ng/µL, 0.05 ng/µL, 0.005 ng/µL, 0.003 ng/µL, and 0.001 ng/µL. In all three replicates of each PCR reaction, we successfully detected and determined the absolute number of the *mex* genes (copies/µL) in the samples with gDNA concentrations ranging from 0.001–2.50 ng/µL (Table 4). Of note, small amounts of background signals detected in the non-template controls led to absolute quantification values lower than 0.5 copies/µL. This result confirmed that the absolute quantification of *mexB*, *mexD*, and *mexY* would decrease with lower gDNA concentrations. The ranges of the absolute quantification of *mexB*, *mexD*, and *mexY* in the samples were 34.81–10,388.27, 15.52–9121.83, and 7.04–5626.67 copies/µL, respectively. Hence, the absolute quantification was attainable from gDNA concentrations of 0.001 ng/µL or higher. As a result, we conclude that the detection limit for the *mex* efflux pump target genes using the *effluxR* detection assay with mdPCR was 0.001 ng/µL. Moreover, there was a good correlation between the gDNA concentrations and the absolute quantification of *mexB*, *mexD*, and *mexY* at R^2^ values of 0.78 (*p* = 0.0035), 0.87 (*p* = 0.0007), and 0.95 (*p* < 0.0001), respectively. We also reported a cut-off value of 3.72 copies/µL for detecting the presence of *mex* efflux genes with the max Youden’s index (J) of 1 (Table 5).

### 3.6. Sensitivity and Specificity of the effluxR Detection Assay with mdPCR System Were 100% for Detecting the Mex Efflux Pump Genes in the P. aeruginosa Strains

**Figure 5:**
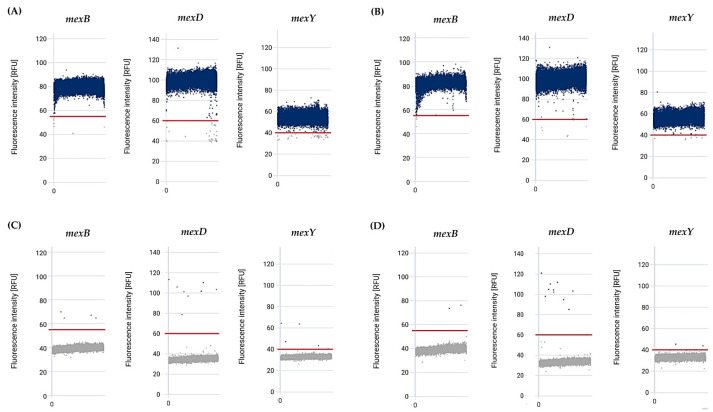
Representative fluorescence intensity of positive-*mex* efflux pump gene samples and negative samples: (**A**) 5 ng/µL of *P. aeruginosa* ATCC27853; (B) 5 ng/µL of *P. aeruginosa* clinical isolate; (**C**) 5 ng/µL of *K. pneumoniae* ATCC1705; and (**D**) non-template. The red lines represent the fluorescence threshold. Blue dots above the threshold line are considered as positive partitions, whereas grey dots below the threshold line are considered as negative partitions.

After determining the limit of detection, a sample-blinded investigation was conducted to evaluate the sensitivity and specificity of the *effluxR* detection assay with the mdPCR system for detecting the *mex* efflux pump genes in *P. aeruginosa* strains. We set up the blinded samples which included 69 known positive samples and 15 known negative samples for the *mex* efflux pump genes. The blinded samples were randomly numbered from Sample 1 to 84 (Table 6). The assay identified the positive partitions of *mexB*, *mexD*, and *mexY* in all positive samples, which is represented as the relative fluorescence intensity in Figure 5. The cut-off value for interpretation was applied among all blinded samples and the non-template control. Using this assay with the mdPCR system, all the *mex* genes, including *mexB*, *mexD*, and *mexY*, were detected in all positive samples (100%), while the *mex* genes were not detected in any negative samples (100%) (Table 6). The blinded investigation showed 100% sensitivity and specificity for the identification of the *mex* efflux pump genes in the *P. aeruginosa* strains. In addition, we did not detect *mexB*, *mexD*, and *mexY* in negative blinded samples from other bacterial strains, further confirming species specificity, namely *S*. *aureus* ATCC29213, *S*. *aureus* ATCC25923, *E*. *coli* ATCC25922, *E*. *coli* ATCC2452, *K*. *pneumoniae* ATCC1705, and *E*. *cloacae* ATCC2341 (Table 6).

## 4. Discussion

The use of efflux pumps is one of the major mechanisms of antibiotic resistance in *P. aeruginosa*, as they facilitate the expulsion of antibiotics or compounds across the outer membrane [18]. The resistance-nodulation-division (RND) efflux pump mechanism constitutes a substantial driver of drug and multidrug resistance in *P*. *aeruginosa* [19]. Notably, antibiotic resistance is primarily influenced by at least three pivotal RND pumps, namely the MexAB-OprM, MexCD-OprJ, and MexXY(-OprA) systems [18,20]. In this work, we sought to detect the presence of *mexB*, *mexD*, and *mexY* genes, which encode efflux pump factors in *P. aeruginosa* strains using a multiplex digital PCR assay. The mdPCR assay can handle multiple targets, making it a valuable tools for large-scale studies and clinical applications [32]. For example, MexA and MexB are parts of the MexAB-OprM efflux pump system, which is responsible for resistance to a broad range of antibiotics, including beta-lactams and fluoroquinolones [33]. MexC and MexD are parts of the MexCD-OprJ efflux pump system, which is related with resistance to fluoroquinolones and other antimicrobials [34]. MexX and MexY are parts of the MexXY-OprM/OprA efflux pump system, which confers resistance to aminoglycosides and other antimicrobial agents [24]. However, there are at least 10 RND-type efflux pump systems of *P. aeruginosa* located in other operons which have yet to be characterized in detail [35]. Here, the three RND systems of greatest significance to antibiotic resistance are studied as they confer resistance to antibiotics including beta-lactams and fluoroquinolones [18,20,21]. Using our *effluxR* detection assay with mdPCR, the presence of these *mex* genes was detected with high specificity in *P. aeruginosa* strains, but not in any of the other bacterial strains investigated. *P. aeruginosa*’s core genome encodes many RND-type efflux pumps, e.g., MexXY, MexAB-OprM, MexCD-OprJ [36], while other microorganism genomes encode other unique types of the RND superfamily efflux pumps, e.g., *E. coli* (AcrAB, AcrAD, AcrEF) [37], *K. pneumoniae* (AcrAB, OqxAB, EefAB, KexD) [38], *E. cloacae* (AcrAB-TolC) [39], *S*. *aureus* (FarE) [40]. According to the high R^2^ values, the absolute quantification of *mexB*, *mexD*, and *mexY* correlates with the gDNA concentrations. As a result, we believe that this assay can be applied to rapidly and accurately detect the presence of *mex* genes in clinical isolates of *P. aeruginosa*. In addition, the *effluxR* detection assay with mdPCR permits detection of genes with gDNA concentrations as low as 0.001 ng/mL (equivalent to 7.04–34.81 copies/µL). Therefore, we propose an appropriate cut-off value of at least 3.72 copies/µL. Absolute quantitative values enable digital PCR results to be used without calibrators or normalisation that would otherwise be required with qPCR [26]. However, the PCR-based technique including qPCR and dPCR has some limitations, such as if the targeted genes contain mutations. If the site of the mutation is located in the primer binding sites, the ability to generate an amplicon would potentially be impaired. However, other genotypic methods could be performed to detect the PCR products containing mutations such as single-stranded conformational polymorphism (SSCP) analysis or sequencing [41].

Genotypic methods such as PCR, RT-PCR, and digital PCR offer several significant advantages over phenotypic assays for assessing efflux pump genes in bacteria [32,42]. Digital PCR is more sensitive and can be further applied to detect the presence of resistance genes at low levels of efflux pump expression [31], thereby providing insights into the role of efflux pumps in the acquisition of antibiotic resistance. Additionally, mdPCR allows the detection of multiple efflux pump genes in a single reaction. Furthermore, genotypic methods are not dependent on growth-based assays and can be performed directly on bacterial DNA or RNA samples, reducing the time and resources required for analysis [32]. Finally, these genotypic methods are not affected by efflux pump inhibitors, providing more robust and reproducible results compared with phenotypic assays. There are several phenotypic assays for efflux pump activity in bacteria that are employed to determine the presence and functionality of efflux pumps, e.g., ethidium bromide/fluorescent dye accumulation assay, efflux pump inhibitor (EPI) assay, etc. [43,44,45]. Nevertheless, these assays have some limitations and disadvantages. Notably, the ethidium bromide (EtBr) accumulation assay lacks specificity due to EtBr’s status as a general substrate that can be extruded by multiple efflux pumps, thus complicating the differentiation between distinct efflux pump types [40,46]. Of further note, EtBr is a mutagenic and hazardous chemical, posing safety concerns, necessitating stringent safety measures during handling and careful disposal to mitigate exposure risks [47]. Moreover, alterations in fluorescence signals in fluorescent dye accumulation assays may be influenced by factors unrelated to efflux pump activity, such as changes in membrane permeability or metabolic state, potentially leading to erroneous interpretations of efflux pump functionality [48]. Furthermore, the efficacy of EPIs may vary across different efflux pumps, resulting in inconsistent inhibition results and impeding direct comparisons among diverse bacterial strains or species [49].

Use of the *effluxR* detection assay with mdPCR serves as a rapid and quantitative diagnostic test as well as a potential tool for early detection of drug resistance, leading to better clinical outcomes and improved antibiotic stewardship in the management of bacterial infections. We recommend the application of the *effluxR* detection assay with multiplex digital PCR for the identification of RND-type resistance genes for rapid, sensitive, and specific detection of *P. aeruginosa* antibiotic resistance. However, this assay should be further investigated in the clinical samples, e.g., blood, sputum, pus, etc. Detecting one of these resistance genes could be used as a genomic-marker identification for *P. aeruginosa* strains, thereby enabling rapid diagnosis of *P. aeruginosa* infections. Further development of this methodology could also permit further uses such as bacterial or fungal pathogen identification, enzyme-mediated resistance gene detection and expression, porin gene expression analysis, and/or the identification and expression of individual RND-efflux pumps.

## 5. Conclusions

Here we show that an *effluxR* detection assay with mdPCR is a high-performance tool for simultaneously detecting the presence of three different *mex* efflux pump genes (*mexB*, *mexD*, and *mexY*) as indicators of drug resistance in clinical *P. aeruginosa* strains. The optimal conditions for detecting *mexB*, *mexD*, and *mexY* using mdPCR were 40 cycles of denaturation at 95 °C for 15 s and combined annealing/extension at 59 °C for 30 s, while the gDNA concentrations ranged from 0.5–5.0 ng/µL. The gDNA detection limit for this assay for *mexB*, *mexD*, and *mexY* genes was 0.001 ng/µL. We also highlight that the *effluxR* detection assay with mdPCR system had 100% sensitivity and specificity for the gene identification with the cut-off value of greater than or equal to 3.72 copies/µL in a blinded sample test. In conclusion, the *effluxR* detection assay with the mdPCR system is suitable to amplify and detect *mex* genes in *P*. *aeruginosa* strains. In the future, using the *effluxR* detection assay with mdPCR systems to detect *mex* genes has the potential to significantly aid clinical decision making for *P*. *aeruginosa* and reduce inappropriate antibiotic use by rationalizing the choice and dosage of antibiotics for *P*. *aeruginosa* infectious treatment.

## Figures and Tables

**Table 1:** The primer and probe sequences were used in this study.

Name	Oligonucleotide Sequences (5′ to 3′)	PCR Product Size (bp)	References
*mexB*		199	[31]
F_primer	GATAGGCCCATTTTCGCGTGG		
R_primer	CGATCCCGTTCATCTGCTGC		
Probe	(FAM)CGCCTTGGTGATCATGCTCGCG(BHQ1)		
*mexD*		131	[31]
F_primer	TCATCAAGCGGCCGAACTTC		
R_primer	GGTGGCGGTGATGGTGATCTG		
Probe	(HEX)CTGGCCGGCCTGCTGGTCATTTC(BHQ1)		
*mexY*		168	[31]
F_primer	CGCAACTGACCCGCTACAAC		
R_primer	CGGACAGGCGTTCTTCGAAG		
Probe	(Texas Red)CGAAGCCATGCAGGCGATGGAGG(BHQ2)		
*16s rRNA*		225	[31]
F_primer	CATGGCTCAGATTGAACGCTG		
R_primer	GCTAATCCGACCTAGGCTCATC		
Probe	(Cy5)CGAGCGGATGAAGGGAGCTTGCTC(BHQ2)		

**Table 2:** The various annealing/extension temperatures and the cycle threshold (CT) values of each gene were detected in two *P*. *aeruginosa* strains using multiplex qPCR.

Strains	Genes	Cycle Threshold Values in Gradient Annealing/Extension Temperature (°C)					*p*-Value
		58	59	60	61	62	
*P. aeruginosa* ATCC27853	*mexB*	16.50 ± 1.10	16.47 ± 1.13	16.53 ± 1.37	16.48 ± 1.77	16.99 ± 1.89	>0.9999
	*mexD*	16.24 ± 0.95	15.57 ± 0.97	15.99 ± 1.57	16.69 ± 1.04	17.66 ± 1.16	>0.9999
	*mexY*	16.12 ± 0.98	15.38 ± 1.10	15.76 ± 1.87	16.38 ± 1.18	17.31 ± 1.45	>0.9999
	*16S rRNA*	13.17 ± 1.60	13.50 ± 0.33	14.03 ± 0.96	14.56 ± 1.45	14.67 ± 0.42	>0.9999
*P. aeruginosa* ATCC BAA-2108	*mexB*	19.77 ± 0.98	20.61 ± 1.34	20.81 ± 0.80	21.21 ± 0.60	23.17 ± 1.08	>0.9999
	*mexD*	16.87 ± 0.62	16.78 ± 1.21	16.40 ± 0.17	16.20 ± 0.17	16.85 ± 1.50	>0.9999
	*mexY*	16.75 ± 0.33	16.79 ± 1.20	16.38 ± 0.34	16.47 ± 0.27	17.42 ± 0.24	>0.9999
	*16S rRNA*	13.37 ± 0.63	16.99 ± 0.56	17.35 ± 1.38	18.92 ± 0.67	18.58 ± 1.23	>0.9999

**Table 3:** *P*. *aeruginosa* gDNA concentrations and cycle threshold (CT) values for detection of each *mex* gene in two *P*. *aeruginosa* strains using multiplex qPCR.

Strains	Target Genes	The CT Values in the Different gDNA Concentrations				*p*-Value
		5.0 ng/µL	2.5 ng/µL	1.0 ng/µL	0.5 ng/µL	
*P. aeruginosa* ATCC27853	*mexB*	17.97 ± 1.31	18.93 ± 0.92	22.65 ± 0.69	23.07 ± 1.26	>0.9999
	*mexD*	20.56 ± 0.93	19.92 ± 0.92	21.74 ± 0.45	22.50 ± 0.52	>0.9999
	*mexY*	19.39 ± 0.61	18.98 ± 0.68	21.71 ± 0.80	22.17 ± 1.12	>0.9999
	*16S rRNA*	15.49 ± 0.90	17.80 ± 0.69	21.04 ± 0.93	20.80 ± 0.39	>0.9999
*P. aeruginosa* ATCC BAA-2108	*mexB*	20.14 ± 0.24	22.98 ± 0.78	23.86 ± 0.22	27.31 ± 1.21	>0.9999
	*mexD*	21.23 ± 0.59	19.80 ± 0.22	20.92 ± 0.41	22.93 ± 0.91	>0.9999
	*mexY*	19.16 ± 0.95	19.18 ± 0.90	20.61 ± 0.54	20.65 ± 1.54	>0.9999
	*16S rRNA*	17.44 ± 1.20	16.34 ± 0.35	19.61 ± 1.30	20.97 ± 0.61	>0.9999

**Table 4:** Absolute quantification (copies/µL) and 95% confidence interval (95% CI) of representative *mex* efflux pump genes presented in various *P. aeruginosa* ATCC27853 gDNA concentrations.

gDNA Concentration Samples (ng/µL)	mexB *		mexD *		mexY *	
	Copies/µL	95% CI	Copies/µL	95% CI	Copies/µL	95% CI
0.001	34.81 ± 9.00	32.30–37.32	15.52 ± 2.83	13.76–17.28	7.04 ± 1.58	5.81–8.26
0.003	87.43 ± 20.37	83.19–91.66	38.32 ± 9.09	35.55–41.09	14.71 ± 1.32	13.04–16.38
0.005	183.82 ± 12.86	176.52–191.12	70.50 ± 12.04	66.67–74.33	33.00 ± 11.53	30.39–35.60
0.050	1923.07 ± 445.32	1891.72–1954.42	1721.94 ± 79.83	1689.22–1754.65	442.64 ± 198.97	431.44–453.83
0.500	7106.50 ± 44.17	6821.53–7391.47	4820.07 ± 919.77	4698.12–4942.02	2086.37 ± 144.30	2050.90–2121.83
1.250	10,184.83 ± 782.63	8901.54–11,468.11	7729.60 ± 640.49	7350.85–8108.35	4117.87 ± 643.55	4031.39–4204.35
2.500	10,388.27 ± 597.32	9092.85–11,683.68	9121.83 ± 1298.64	8300.86–9942.79	5626.67 ± 733.42	5465.75–5787.59
NTC	0.04 ± 0.03	−0.02–0.09	0.40 ± 0.08	0.085–0.72	0.30 ± 0.04	0.06–0.53

* Note: R^2^ values between the gDNA concentrations and the absolute quantification of *mexB*, *mexD*, and *mexY* were 0.78 (*p* = 0.0035), 0.87 (*p* = 0.0007), and 0.95 (*p* < 0.0001), respectively.

**Table 5:** The cut-off values of the *effluxR* detection assay with mdPCR using ROC analysis and Youden’s index.

Cut-Off Values of the Copy Number of Genes (µg/mL)	Sensitivity	Specificity	1—Specificity	Youden’s Index (J)
−0.960	1.000	0.000	1.000	0.000
0.170	1.000	0.333	0.667	0.333
0.350	1.000	0.667	0.333	0.667
3.720	1.000	1.000	0.000	1.000
10.875	0.952	1.000	0.000	0.952
15.115	0.905	1.000	0.000	0.905
24.260	0.857	1.000	0.000	0.857
33.905	0.810	1.000	0.000	0.810
36.565	0.762	1.000	0.000	0.762
54.410	0.714	1.000	0.000	0.714
78.965	0.667	1.000	0.000	0.667
135.625	0.619	1.000	0.000	0.619
313.230	0.571	1.000	0.000	0.571
1082.290	0.524	1.000	0.000	0.524
1822.505	0.476	1.000	0.000	0.476
2004.720	0.429	1.000	0.000	0.429
3102.120	0.381	1.000	0.000	0.381
4468.970	0.333	1.000	0.000	0.333
5223.370	0.286	1.000	0.000	0.286
6366.585	0.238	1.000	0.000	0.238
7418.050	0.190	1.000	0.000	0.190
8425.715	0.143	1.000	0.000	0.143
9653.330	0.095	1.000	0.000	0.095
10,286.550	0.048	1.000	0.000	0.048
10,389.270	0.000	1.000	0.000	0.000

**Table 6:** The *effluxR* detection assay with mdPCR detected the 69 positive samples from 84 blinded bacterial reference and clinical isolate strains. No positive signal for *mex* genes was detected in the 15 negative samples.

Sample No.	Bacterial Species	Present of *Mex* Genes			Result of *effluxR* Detection Assay
		*mexB*	*mexD*	*mexY*	
1	*P. aeruginosa*	+	+	+	Positive for three genes
2	*P. aeruginosa*	+	+	+	Positive for three genes
3	*E. coli*	-	-	-	Negative for three genes
4	*P. aeruginosa*	+	+	+	Positive for three genes
5	*P. aeruginosa*	+	+	+	Positive for three genes
6	*P. aeruginosa*	+	+	+	Positive for three genes
7	*S. aureus*	-	-	-	Negative for three genes
8	*E. cloacae*	-	-	-	Negative for three genes
9	*P. aeruginosa*	+	+	+	Positive for three genes
10	*P. aeruginosa*	+	+	+	Positive for three genes
11	*P. aeruginosa*	+	+	+	Positive for three genes
12	*P. aeruginosa*	+	+	+	Positive for three genes
13	*E. cloacae*	-	-	-	Negative for three genes
14	*P. aeruginosa*	+	+	+	Positive for three genes
15	*P. aeruginosa*	+	+	+	Positive for three genes
16	*P. aeruginosa*	+	+	+	Positive for three genes
17	*P. aeruginosa*	+	+	+	Positive for three genes
18	*P. aeruginosa*	+	+	+	Positive for three genes
19	*P. aeruginosa*	+	+	+	Positive for three genes
20	*K. pneumoniae*	-	-	-	Negative for three genes
21	*P. aeruginosa*	+	+	+	Positive for three genes
22	*P. aeruginosa*	+	+	+	Positive for three genes
23	*P. aeruginosa*	+	+	+	Positive for three genes
24	*P. aeruginosa*	+	+	+	Positive for three genes
25	*P. aeruginosa*	+	+	+	Positive for three genes
26	*P. aeruginosa*	+	+	+	Positive for three genes
27	*P. aeruginosa*	+	+	+	Positive for three genes
28	*S. aureus*	-	-	-	Negative for three genes
29	*P. aeruginosa*	+	+	+	Positive for three genes
30	*P. aeruginosa*	+	+	+	Positive for three genes
31	*S. aureus*	-	-	-	Negative for three genes
32	*P. aeruginosa*	+	+	+	Positive for three genes
33	*P. aeruginosa*	+	+	+	Positive for three genes
34	*P. aeruginosa*	+	+	+	Positive for three genes
35	*E. coli*	-	-	-	Negative for three genes
36	*P. aeruginosa*	+	+	+	Positive for three genes
37	*P. aeruginosa*	+	+	+	Positive for three genes
38	*P. aeruginosa*	+	+	+	Positive for three genes
39	*P. aeruginosa*	+	+	+	Positive for three genes
40	*K. pneumoniae*	-	-	-	Negative for three genes
41	*P. aeruginosa*	+	+	+	Positive for three genes
42	*P. aeruginosa*	+	+	+	Positive for three genes
43	*P. aeruginosa*	+	+	+	Positive for three genes
44	*P. aeruginosa*	+	+	+	Positive for three genes
45	*E. coli*	-	-	-	Negative for three genes
46	*K. pneumoniae*	-	-	-	Negative for three genes
47	*P. aeruginosa*	+	+	+	Positive for three genes
48	*P. aeruginosa*	+	+	+	Positive for three genes
49	*P. aeruginosa*	+	+	+	Positive for three genes
50	*P. aeruginosa*	+	+	+	Positive for three genes
51	*P. aeruginosa*	+	+	+	Positive for three genes
52	*P. aeruginosa*	+	+	+	Positive for three genes
53	*P. aeruginosa*	+	+	+	Positive for three genes
54	*P. aeruginosa*	+	+	+	Positive for three genes
55	*P. aeruginosa*	+	+	+	Positive for three genes
56	*P. aeruginosa*	+	+	+	Positive for three genes
57	*P. aeruginosa*	+	+	+	Positive for three genes
58	*S. aureus*	-	-	-	Negative for three genes
59	*P. aeruginosa*	+	+	+	Positive for three genes
60	*P. aeruginosa*	+	+	+	Positive for three genes
61	*P. aeruginosa*	+	+	+	Positive for three genes
62	*P. aeruginosa*	+	+	+	Positive for three genes
63	*P. aeruginosa*	+	+	+	Positive for three genes
64	*P. aeruginosa*	+	+	+	Positive for three genes
65	*P. aeruginosa*	+	+	+	Positive for three genes
66	*P. aeruginosa*	+	+	+	Positive for three genes
67	*P. aeruginosa*	+	+	+	Positive for three genes
68	*E. coli*	-	-	-	Negative for three genes
69	*P. aeruginosa*	+	+	+	Positive for three genes
70	*P. aeruginosa*	+	+	+	Positive for three genes
71	*P. aeruginosa*	+	+	+	Positive for three genes
72	*P. aeruginosa*	+	+	+	Positive for three genes
73	*P. aeruginosa*	+	+	+	Positive for three genes
74	*K. pneumoniae*	-	-	-	Negative for three genes
75	*P. aeruginosa*	+	+	+	Positive for three genes
76	*P. aeruginosa*	+	+	+	Positive for three genes
77	*K. pneumoniae*	*-*	-	-	Negative for three genes
78	*P. aeruginosa*	+	+	+	Positive for three genes
79	*P. aeruginosa*	+	+	+	Positive for three genes
80	*P. aeruginosa*	+	+	+	Positive for three genes
81	*P. aeruginosa*	+	+	+	Positive for three genes
82	*P. aeruginosa*	+	+	+	Positive for three genes
83	*P. aeruginosa*	+	+	+	Positive for three genes
84	*P. aeruginosa*	+	+	+	Positive for three genes
	Total	69	69	69	
	Percentage	100	100	100	

Note: symbol (+) is a present gene and (-) is an absent gene.

## Data Availability

The data supporting the current study are available from the corresponding author upon request.
